# An amphiphilic *pseudo*[1]catenane: neutral guest-induced clouding point change

**DOI:** 10.3762/bjoc.14.167

**Published:** 2018-07-26

**Authors:** Tomoki Ogoshi, Tomohiro Akutsu, Tada-aki Yamagishi

**Affiliations:** 1 Graduate School of Natural Science and Technology, Kanazawa University, Kakuma-machi, Kanazawa 920-1192, Japan; 2 WPI Nano Life Science Institute, Kanazawa University, Kakuma-machi, Kanazawa 920-1192, Japan

**Keywords:** amphiphilic molecules, host–guest complexes, lower critical solution temperature, pillar[*n*]arenes, *pseudo*[1]catenane

## Abstract

The hydrophobic/hydrophilic ratio in a molecule largely affects its assembled properties in aqueous media. In this study, we synthesized a new bicyclic compound which could dynamically change its hydrophobic/hydrophilic ratio by chemical stimulus. The bicyclic compound consisted of amphiphilic pillar[5]arene and hydrophobic alkyl chain rings, and formed a self-inclusion structure in aqueous media, which was assigned as a *pseudo*[1]catenane structure. The hydrophobic chain ring was hidden inside the pillar[5]arene cavity in the *pseudo*[1]catenane structure, thus the bicyclic compound was soluble in water at 20 °C with a clouding point at 24 °C. The *pseudo*[1]catenane was converted to the de-threaded structure upon addition of the neutral guest 1,4-dicyanobutane, which displaced the alkyl chain ring from the inside to the outside of the cavity. The hydrophobic alkyl chain ring was now exposed to the aqueous media, causing aggregation of the hydrophobic alkyl chain rings, which induced insolubilization of the bicyclic compound in aqueous media at 20 °C and a decrease in its clouding point.

## Introduction

**Figure 1:**
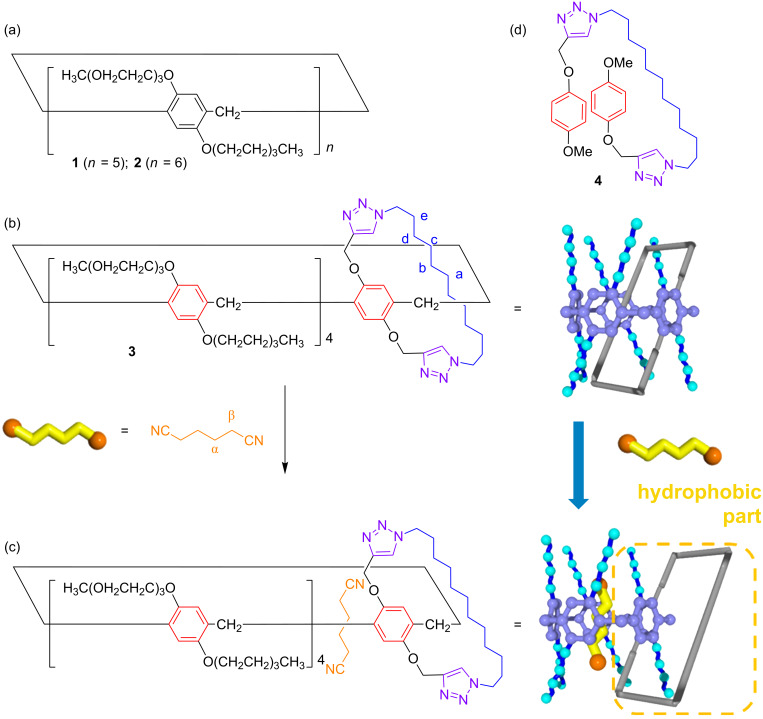
Chemical structures of (a) tri(ethylene oxide)-substituted pillar[*n*]arenes (**1**, *n* = 5; **2**, *n* = 6), (b) *pseudo*[1]catenane **3**, (c) de-threaded form of **3** by complexation with 1,4-dicyanobutane, and (d) model compound **4**.

Thermo-responsive molecules exhibiting a lower critical solution temperature (LCST) are very important for applications such as controlled drug release [1], molecular separation [2], and tissue culture substrates [3]. Poly(*N*-isopropylacrylamide) (pNIPAAm) is a widely used thermo-responsive polymer, which exhibits a clouding point around 32 °C [1–4]. Recently, thermo-responsive molecules with additional functions have been developed to replace pNIPAAm [5–10]. For example, we have developed thermo-responsive macrocyclic molecules which exhibit LCST behavior regulated by host–guest chemistry [5–7]. The molecules consist of a non-ionic amphiphilic part containing tri(ethylene oxide) moieties, and a hydrophobic part consisting of a pillar[*n*]arene core (Figure 1a; **1**, *n* = 5; **2**, *n* = 6).

Pillar[*n*]arenes, which were first reported by our group [11], were used as the macrocyclic component because of their high functionality and superior host–guest properties with neutral guests [12–16]. The amphiphilic pillar[*n*]arenes **1** and **2** exhibit clouding points at 41 and 42 °C, respectively [5–6]. The oligo(ethylene oxide) moieties are solvated with water molecules at room temperature, but de-solvation occurs upon heating, which triggers aggregation of the hydrophobic pillar[*n*]arene cores. The amphiphilic pillar[*n*]arenes can capture guest molecules with encapsulation of an ionic guest molecule resulting in a change of the hydrophobic/hydrophilic ratio, and consequently a change in the clouding point. The clouding point of **1** increased upon addition of a cationic guest as the hydrophilic ratio in the molecule increased by formation of the complex with the hydrophilic cationic guest [5]. We also demonstrated photoresponsive LCST behavior by using a photoresponsive host–guest complex system between amphiphilic pillar[6]arene **2** and a photoresponsive cationic azobenzene guest [6]. However, neutral guest molecules could not induce a clear LCST change because encapsulation of the neutral guest in the hydrophobic pillar[*n*]arene core did not significantly change the hydrophobic/hydrophilic ratio. In this study, we successfully induced an LCST change using a neutral guest and a new pillar[5]arene derivative. We synthesized a new bicyclic compound consisting of an amphiphilic pillar[5]arene and hydrophobic alkyl chain rings **3** (Figure 1b). This bicyclic compound formed a self-inclusion structure in aqueous media, which was assigned as a *pseudo*[1]catenane structure. The hydrophobic alkyl chain ring was hidden in the pillar[5]arene cavity in the *pseudo*[1]catenane structure, thus **3** was soluble in water at 20 °C and exhibited a clouding point at 24 °C. However, a de-threaded structure formed upon addition of a neutral guest, 1,4-dicyanobutane. The supramolecular structural change contributed to a significant increase in the hydrophobic ratio of the molecule, which induced insolubilization of **3** in aqueous media at 20 °C and a decrease of the clouding point temperature. Neutral guest-responsive LCST changes are very rare, while there have been some examples of LCST control using ionic chemical stimuli [5–6].

## Results and Discussion

### Supramolecular structure and clouding point of bicyclic compound **3**

**Figure 2:**
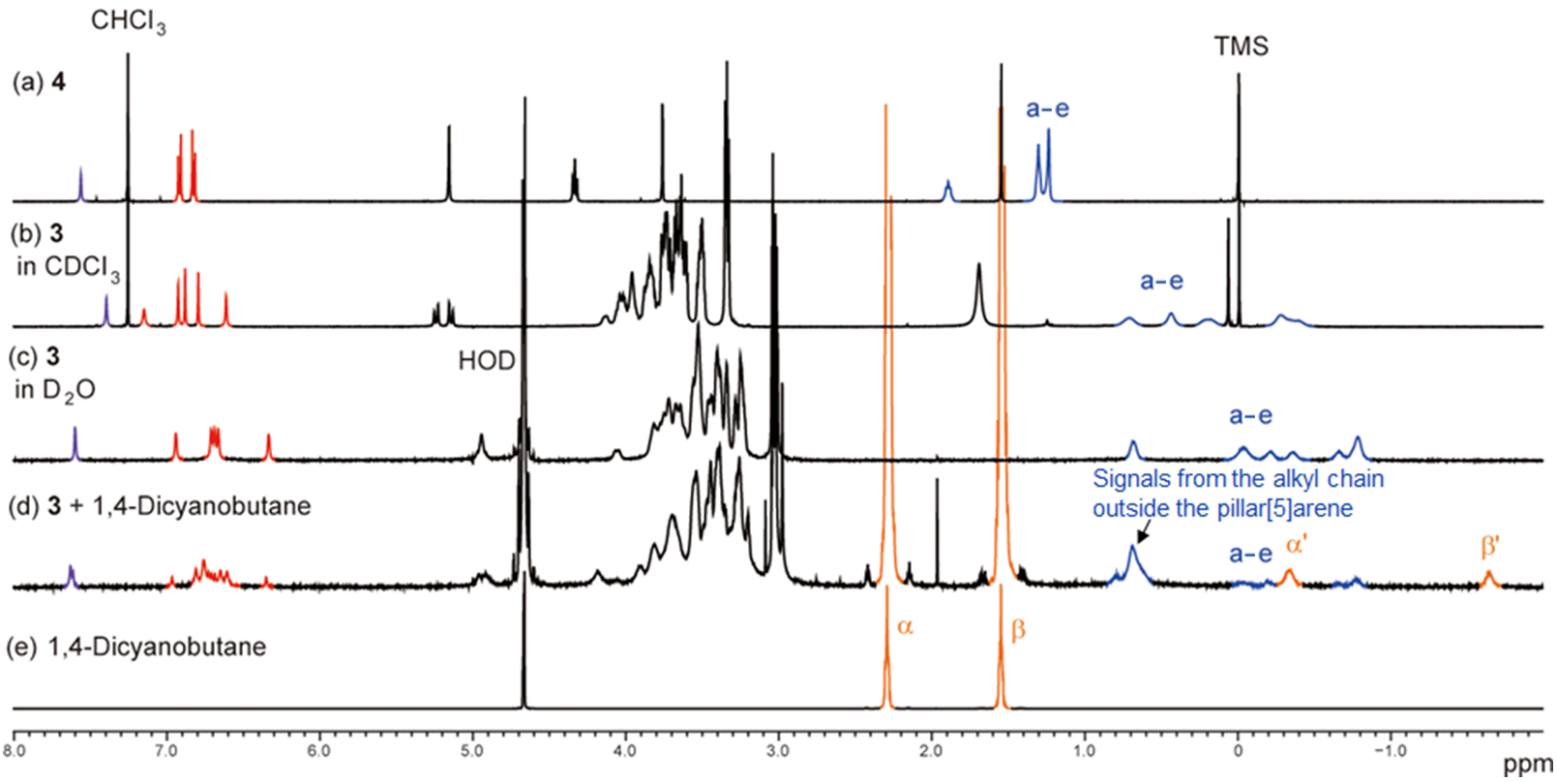
^1^H NMR spectra of (a) model compound **4** (2 mM at 25 °C in CDCl_3_), (b) **3** (2 mM at 25 °C in CDCl_3_), (c) **3** (2 mM at 10 °C in D_2_O), (d) **3** (2 mM) and 1,4-dicyanobutane (20 mM) at 10 °C in D_2_O and (e) 1,4-dicyanobutane (20 mM at 10 °C in D_2_O). Resonances are labeled as shown in Figure 1.

The bicyclic compound **3** was prepared using a copper(I)-catalyzed alkyne–azide cycloaddition (CuAAC) ‘‘click’’ reaction (see details in the experimental section). In addition, model compound **4** was also synthesized as a reference (Figure 1d). Compound **3** is soluble in various organic and aqueous solvents as it comprises eight amphiphilic tri(ethylene oxide) chains. We investigated the supramolecular structure of **3** by ^1^H NMR spectroscopy. In CDCl_3_, the signals from the alkyl chain of **3** (blue peaks, a–e, Figure 2) were observed upfield compared with the ones of the model compound **4** (Figure 2a). This is due to an aromatic shielding by the pillar[5]arene cavity, indicating that **3** mainly formed a *pseudo*[1]catenane structure in CDCl_3_. The linear alkyl chains can act as guests for pillar[5]arenes, thus the *pseudo*[1]catenane structure was stable in CDCl_3_ [17–19]. In D_2_O, as in CDCl_3_, the proton signals from the alkyl chain ring (blue peaks, a–e) were also observed upfield (Figure 2c), indicating the formation of a *pseudo*[2]catenane structure in D_2_O. The alkyl chain ring not only acts as a good guest for the pillar[5]arene, but also as a hydrophobic portion, thus the *pseudo*[2]catenane structure would be favored in D_2_O.

**Figure 3:**
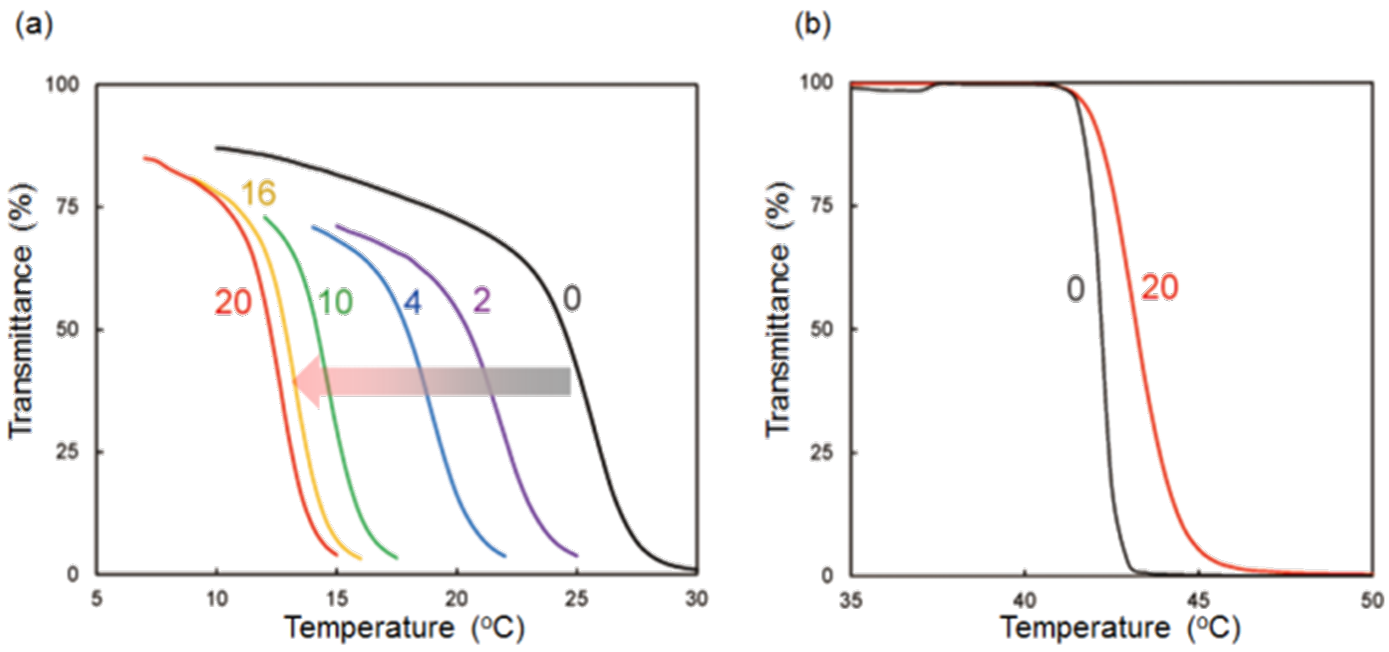
Temperature dependence of light transmittance at 650 nm of an aqueous solution of (a) **3** (2 mM) upon addition of 1,4-dicyanobutane (0–20 mM) and (b) **1** (2 mM) in the presence and absence of 1,4-dicyanobutane (20 mM) on heating.

As for pillar[5]arene **1** with 10 tri(ethylene oxide) chains, compound **3** also exhibits LCST behavior. Compound **3** is soluble in aqueous media at 20 °C, the solution becomes turbid at 40 °C, and turns back to a clear solution upon cooling. The clouding point of **3** was determined by monitoring the transmittance change (Figure 3). The clouding point of **3**, determined by monitoring the transmittance change, was 24 °C (2 mM in aqueous solution, Figure 3a, black line) which is 18 °C lower than the clouding point of **1** (42 °C, Figure 3b, black line) [5]. This is due to the fact that the benzene units carrying the hydrophobic alkyl chain ring in **3** are more hydrophobic than the tri(ethylene oxide)-substituted benzene unit in **1**, although the hydrophobic alkyl chain ring was hidden inside the cavity by formation of the *pseudo*[1]catenane structure in aqueous media.

### Effect of a supramolecular structural change on the clouding point

To displace the alkyl chain ring of **3** from the inside to the outside of the cavity, a competitive guest molecule, 1,4-dicyanobutane, was added. 1,4-Dicyanobutane was chosen because it forms highly stable 1:1 host–guest complexes with pillar[5]arenes (*K* > 10^4^ M^−1^) [20]. Figure 2d shows the ^1^H NMR spectra of **3** in the presence of 1,4-dicyanobutane. In the spectrum, additional peaks were observed upon the addition of 1,4-dicyanobutane (orange peaks α’ and β’) in Figure 2d. For comparison Figure 2e shows the spectrum of 1,4-dycanaobutane (orange peaks α and β). Similar signals were also observed in the host–guest complexes of 1,4-dicyanobutane with other pillar[5]arenes [17,20], and were assigned as the proton signals from the methylene protons of the 1,4-dicyanobutane in the cavity of pillar[5]arene **3**. The complex formation of **3** and 1,4-dicyanobutane indicates the displacement of the alkyl chain ring from the inside to the outside of the cavity by 1,4-dicyanobutane. The association constant between the *pseudo*[1]catenane structure **3** and 1,4-dicyanobutane estimated by ^1^H NMR at 25 °C was 13.9 M^−1^ (Supporting Information File 1, Figure S6), which is remarkably lower than the association constant between **1** and 1,4-dicyanobutane (4.6 × 10^4^ M^−1^, Figure S8 in Supporting Information File 1). The *pseudo*[1]catenane structure is very stable, thus the alkyl chain ring acts as a competitive guest to 1,4-dicyanobutane.

The effect of the supramolecular structural change from the *pseudo*[1]catenane to the de-threaded structure on the clouding point change was investigated next. Even at a low concentration of the competitive guest 1,4-dicyanobutane (2 mM), the clouding point clearly decreased, indicating de-threading of the hydrophobic alkyl chain extremely contributed to the change in the hydrophobic/hydrophilic ratio. With increasing concentrations of 1,4-dicyanobutane, the clouding points gradually decreased from 24 °C to 12 °C (Figure 3a). Formation of the de-threaded form by complexation between **3** and 1,4-dicyanobutane induced aggregation of the alkyl chain ring on the outside of the cavity. However, only a very minor change in the clouding point was observed upon addition of 1,4-dicyanobutane to an aqueous solution of **1** (Figure 3b, indicating that the hydrophobic/hydrophilic ratio did not change much by the host–guest complexation between **1** and 1,4-dicyanobutane because 1,4-dicyanobutane was hidden inside the hydrophilic pillar[5]arene cavity. Therefore, the supramolecular structural change of **3** induced a dramatic change in its hydrophobic/hydrophilic ratio, and consequently the clouding point change. Based on the supramolecular structural change, the neutral guest, 1,4-dicyanobutane can be used for changing the clouding point of **3**.

**Figure 4:**
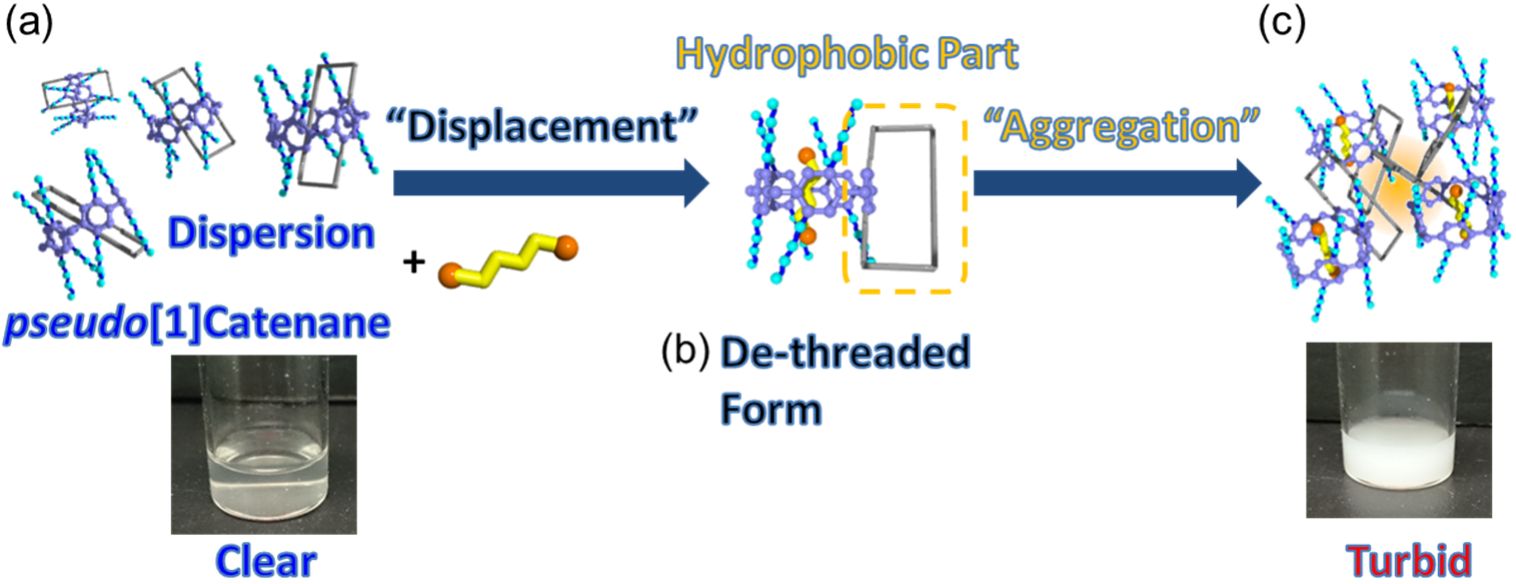
Photographs of (a) **3** and (c) a mixture of **3** (2 mM) and 1,4-dicyanobutane (20 mM) in aqueous media at 20 °C and schematic representation of neutral-guest responsive clouding point change using supramolecular structural change from *pseudo*[1]catenane to de-threaded form upon (b) addition of a competitive guest, 1,4-dicyanobutane.

We then demonstrated a chemically responsive transmittance change using the supramolecular structural change. An aqueous solution containing **3** at 20 °C was clear (Figure 4a) as the clouding point of the self-inclusion structure **3** is 24 °C. Upon addition of the neutral guest 1,4-dicyanobutane, the solution changed from clear to turbid (Figure 4c) as the clouding point of the complex is 12 °C.

The details of this neutral guest-responsive LCST change are summarized in Figure 4. Compound **3** formed a *pseudo*[1]catenane structure in aqueous media (Figure 4a), with the water-insoluble hydrophobic part of the alkyl chain ring hidden inside the hydrophilic pillar[5]arene cavity, meaning that **3** was soluble in water. The clear solution of **3** became turbid upon addition of the competitive neutral guest 1,4-dicyanobutane. The competitive guest displaced the hydrophobic alkyl chain ring from the inside to the outside of the cavity (Figure 4b), causing aggregation of the hydrophobic alkyl chain ring by hydrophobic interactions (Figure 4c).

## Conclusion

We have successfully prepared a new amphiphilic bicyclic compound **3**. A key feature of the molecule is that the hydrophobic part of the alkyl chain ring is hidden by formation of a *pseudo*[1]catenane structure. The movement of the alkyl chain ring from the inside to the outside of the cavity upon the addition of the competitive guest molecule dramatically contributed to the clouding point change. A neutral guest molecule, 1,4-dicyanobutane, was able to induce a change in the clouding point via a supramolecular structural change from a *pseudo*[1]catenane to the de-threaded form. Bicyclic structures have previously been used to induce color changes by adding guests, and are also referred to as “molecular chameleons” and “catenane-chameleons” [21–22]. However, to the best of our knowledge, the use of a dynamic supramolecular structural change of **3** to induce an LCST change is the first example in this area. Thus, this work may open the way for the design of a new generation of molecular assembly systems using supramolecular structures such as rotaxane, catenanes, polyrotaxanes and polycatenanes.

## Experimental

**Materials.** All solvents and reagents were used as supplied. Compounds **1** and **4** were synthesized according to previous reported procedures [5,17].

**Measurements.** The ^1^H NMR spectra were recorded at 500 MHz and ^13^C NMR spectra were recorded at 125 MHz with a JEOL-ECA500 spectrometer. UV–vis absorption spectra were recorded with a JASCO V-670 using 1 cm quartz cuvettes. Cloud points were determined by transmission changes (at 650 nm) of the solutions heated at 0.1 °C/min; cloud point values were defined as the temperature at which the transmission decreases by 50% [23].

**Scheme 1:**
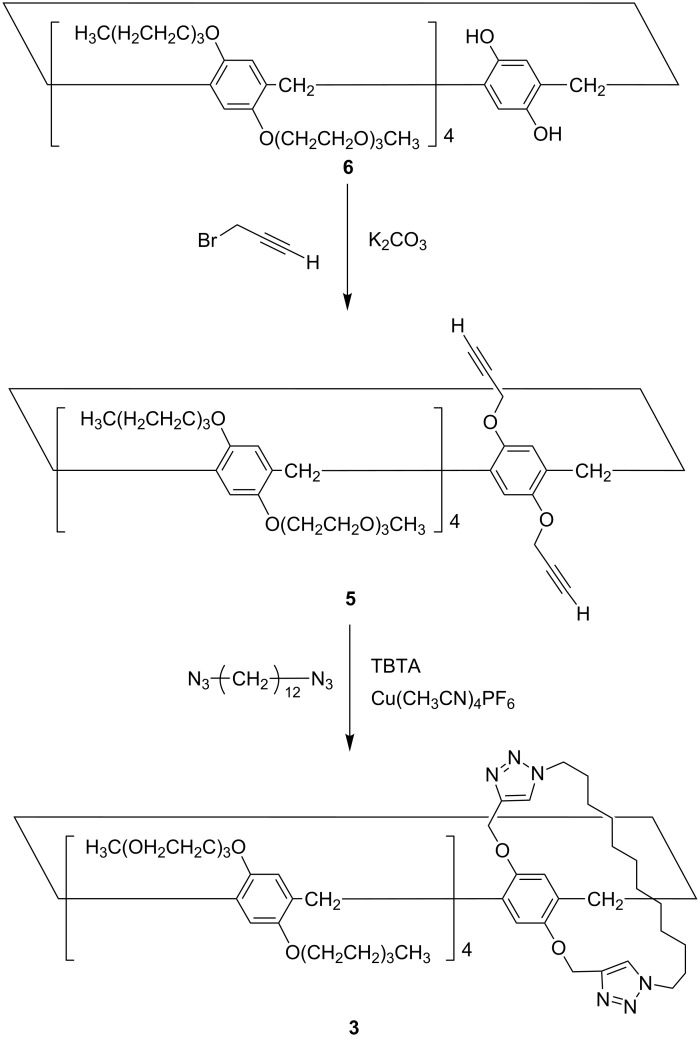
Synthesis of the bicyclic compound **3**.

**Pillar[5]arene carrying 2 alkyne groups on the same unit (5).** In a similar manner as described in [24], **5** was prepared. Under a nitrogen atmosphere, pillar[5]arene carrying one hydroquinone unit **6** [7] (Scheme 1, 417 mg, 0.230 mmol) was dissolved in acetone (10 mL). K_2_CO_3_ (159 mg, 1.15 mmol) was added and the reaction mixture was stirred. Then, propargyl bromide (2.30 g, 2.30 mmol) was added and the reaction mixture was heated at 80 °C for 24 h. The reaction mixture was cooled to room temperature and the precipitate was removed by filtration. After the filtration, the solvents were evaporated to afford a solid. Column chromatography (silica gel; methanol/ethyl acetate 1:9) afforded **5** as a solid (298 mg, 0.161 mmol, yield: 70%). ^1^H NMR (500 MHz, CDCl_3_) δ 6.88, 6.84, 6.83, 6.81, 6.78 (s, 10H, phenyl), 4.53 (d, 4H, methylene), 4.00, 3.42–3.83 (m, 106H, methylene), 3.27–3.39 (m, 24H, methyl), 2.27 (t, 2H, alkyne) ppm; ^13^C NMR (125 MHz, CDCl_3_) δ 150.0, 149.8, 149.3, 129.1, 129.0, 128.6, 128.4, 115.6, 115.4, 115.2, 115.1, 71.9, 70.9, 70.8, 70.6, 70.4, 70.3, 68.3, 68.2, 68.1, 68.0, 59.0, 56.5, 29.7, 29.3 ppm; HRMS–ESI (*m*/*z*): [M + Na]^+^ calcd for C_97_H_146_NaO_34_, 1877.9593; found, 1877.9612.

**Bicyclic compound 3.** In a similar manner as described in [17], we synthesized bicyclic compound **3**. Tris[(1-benzyl-1*H*-1,2,3-triazol-4-yl)methyl]amine (31.8 mg, 60.0 μmol) and [Cu(CH_3_CN)_4_]PF_6_ (22.3 mg, 60.0 μmol) were added to a solution containing **5** (111 mg, 60.0 μmol) and 1,12-diazidododecane (15.1 mg, 60.0 μmol) in dichloromethane (300 mL), and the mixture was stirred at 25 °C for 12 h. The resulting solution was concentrated in vacuo. Column chromatography (silica gel, ethyl acetate/methanol 1:1) afforded the bicyclic compound **3** (12.6 mg, 6.00 μmol, yield: 10%).^1^H NMR (500 MHz, CDCl_3_) δ 7.40 (s, 2H, triazole), 7.15 (s, 2H, phenyl), 6.93 (s, 2H, phenyl), 6.89 (s, 2H, phenyl), 6.80 (s, 2H, phenyl), 6.62 (s, 2H, phenyl), 5.21 (dd, 4H, methylene), 3.50–4.14 (m, 110H, methylene), 3.34–3.36 (m, 24H, methyl), −0.27–0.72 (br, 20H, methylene of alkyl chain) ppm; ^13^C NMR (125 MHz, CDCl_3_) δ 150.4, 149.8, 149.7, 149.6, 149.4 129.0, 128.6, 128.3, 124.3, 115.7, 115.0, 71.9, 70.8, 70.7, 70.6, 70.3, 68.4, 68.1, 67.8, 67.6, 59.0, 29.7, 29.0, 28.8, 28.5, 27.3, 24.5 ppm; HRMS–ESI (*m*/*z*): [M + 2Na]^2+^ calcd for C_109_H_170_N_6_Na_2_O_34_, 1076.5777; found, 1076.5635.

**Determination of association constants.** In a similar manner as described in [6], association constants of the host–guest complexes were determined. In the host–guest complex of 1,4-dicyanobutane with **3** in D_2_O, the chemical exchange between the complexed and uncomplexed species was slow on the NMR timescale. Thus, the ^1^H NMR spectra of mixtures of **3** with 1,4-dicyanobutane showed two sets of resonances for complexed (Figure 2d, peaks α’ and β’) and uncomplexed 1,4-dicyanobutane (peaks, α and β). The association constant for the host–guest complexes between 1,4-dicyanobutane and **3** at 10 °C was 24.5 M^−1^, which was calculated from integrations of the complexed (Supporting Information File 1, Figure S5, orange peak β’) and uncomplexed signals (Supporting Information File 1, Figure S5, orange peak β) of 1,4-dicyanobutane. The association constant of the complex at 25 °C extrapolated by van 't Hoff plots (Figure S6 in the Supporting Information File 1) was 13.9 M^−1^.

In the host–guest complex of 1,4-dicyanobutane with **1** in D_2_O, chemical exchange between the complexed and uncomplexed species was also slow on the NMR timescale. Thus, ^1^H NMR spectra of mixtures of **1** with 1,4-dicyanobutane showed two sets of resonances for complexed (Figure 2d, peaks α’ and β’) and uncomplexed 1,4-dicyanobutane (peaks, α and β). The association constant for the host–guest complex of 1,4-dicyanobutane and **1** at 25 °C was 4.7 × 10^4^ M^−1^, which was calculated from the integrations of the complexed (Supporting Information File 1, Figure S7, orange peak β') and uncomplexed signals (Supporting Information File, Figure S7, orange peak β) of 1,4-dicyanobutane.

## Supporting Information

File 1 ^1^H and ^13^C NMR spectra of **3** and **5**, variable temperature ^1^H NMR spectra of a mixture of **3** and **5** with 1,4-dicyanobutane and van 't Hoff plots.
