# Supplementary material for: An amphiphilic pseudo[1]catenane: neutral guest-induced clouding point change
Source: Beilstein J Org Chem. 2018 Jul 26;14:1937–43. doi: 10.3762/bjoc.14.167 (PMC6071686; doi:10.3762/bjoc.14.167)
Supplement: File 1 — 1H and 13C NMR spectra of 3 and 5, variable temperature 1H NMR spectra of a mixture of 3 and 5 with 1,4-dicyanobutane and van 't Hoff plots. [file Beilstein_J_Org_Chem-14-1937-s001.pdf]

# Supporting Information

# for

# An amphiphilic *pseudo*[1]catenane: neutral guest-induced clouding point change

Tomoki Ogoshi*[1,2], Tomohiro Akutsu[1] and Tada-aki Yamagishi[1]

Address: [1]Graduate School of Natural Science and Technology, Kanazawa University, Kakuma-machi, Kanazawa 920-1192, Japan and [2]WPI Nano Life Science Institute, Kanazawa University, Kakuma-machi, Kanazawa 920-1192, Japan

Email: Tomoki Ogoshi - ogoshi@se.kanazawa-u.ac.jp

*Corresponding author

## $^{1}$H and $^{13}$C NMR spectra of **3** and **5**, variable temperature $^{1}$H NMR spectra of a mixture of **3** and **5** with 1,4-dicyanobutane and van 't Hoff plots

**Table of Contents**

Figure S1: $^{1}$H NMR spectrum of **5** ........ S2
Figure S2: $^{13}$C NMR spectrum of **5** ........ S2
Figure S3: $^{1}$H NMR spectrum of **3** ........ S3
Figure S4 :$^{13}$C NMR spectrum of **3** ........ S3
Figure S5: Variable temperature $^{1}$H NMR spectra of a mixture of **3** and 1,4-dicyanobutane........ S4
Figure S6: van 't Hoff plots for a mixture of **3** and 1,4-dicyanobutane........ S4
Figure S7: Variable Temperature $^{1}$H NMR spectra of a mixture of **1** and 1,4-dicyanobutane ........ S5
Figure S8: van 't Hoff plots for a mixture of **1** and 1,4-dicyanobutane........ S5

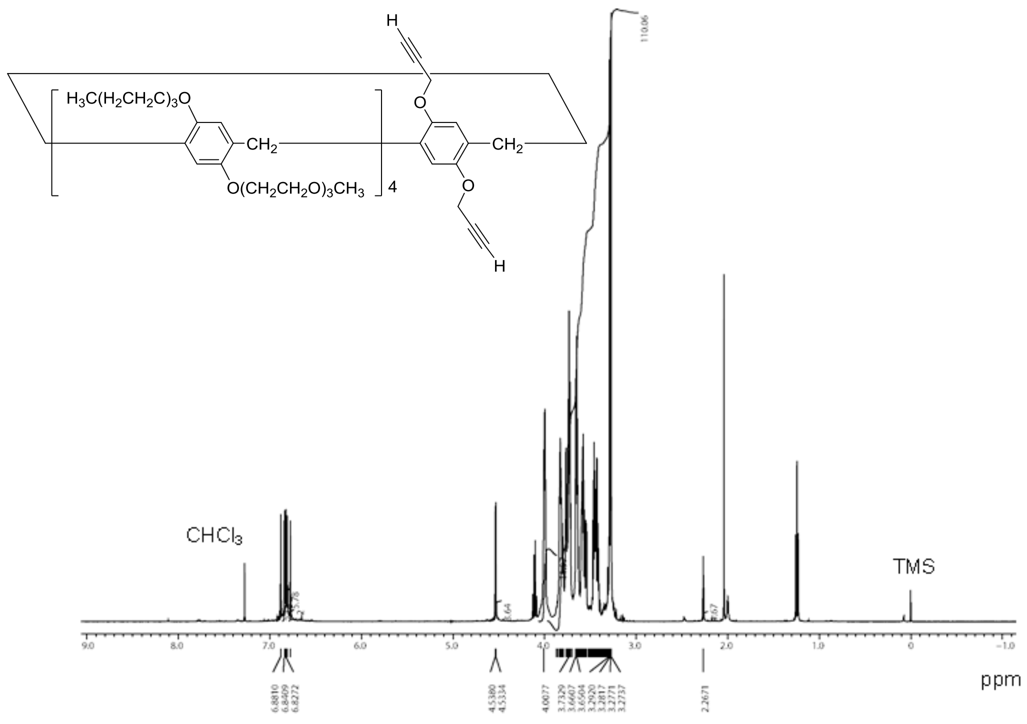


**Figure S1:** $^{1}$H NMR spectrum ($CDCl_3$, 25 °C) of **5**.

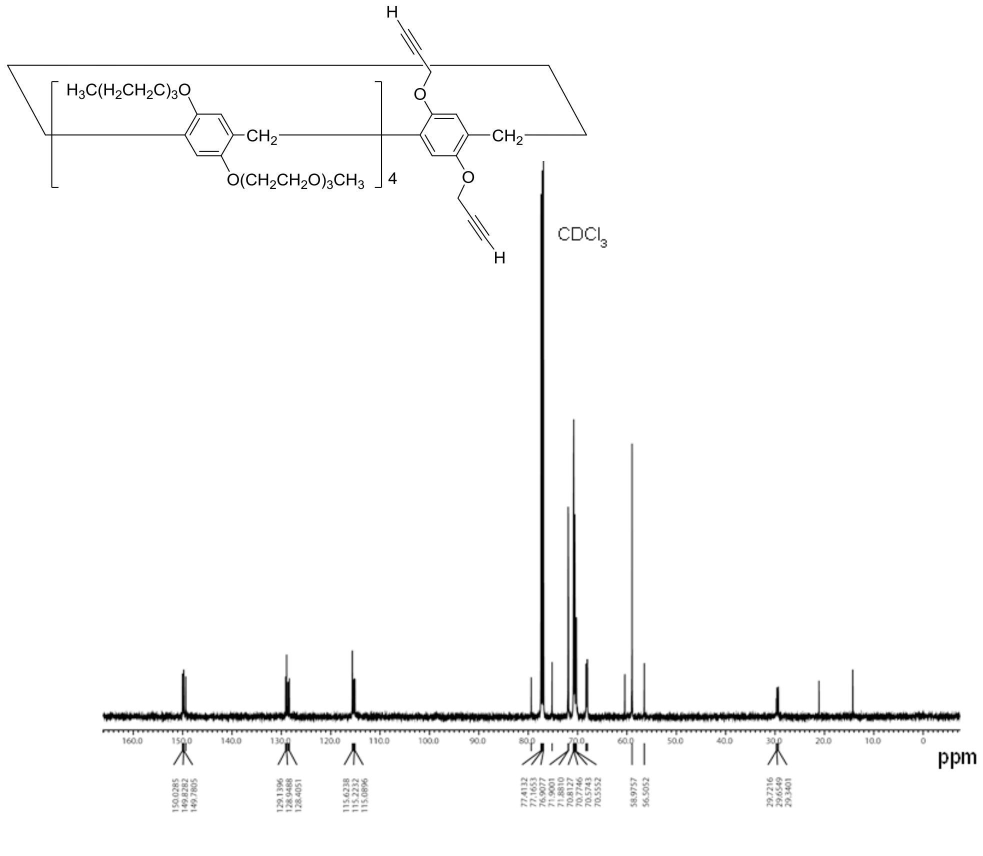


**Figure S2:** $^{13}$C NMR spectrum ($CDCl_3$, 25 °C) of **5**.

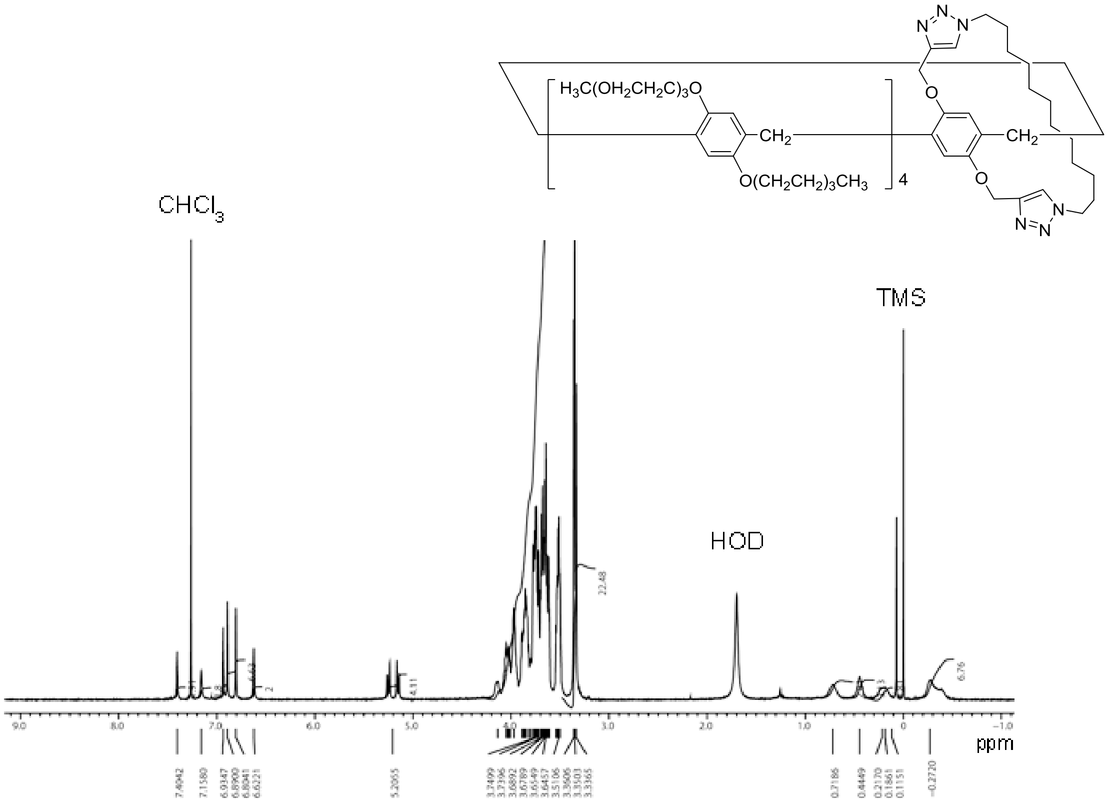


**Figure S3:** $^{1}$H NMR spectrum ($CDCl_3$, 25 °C) of **3**.

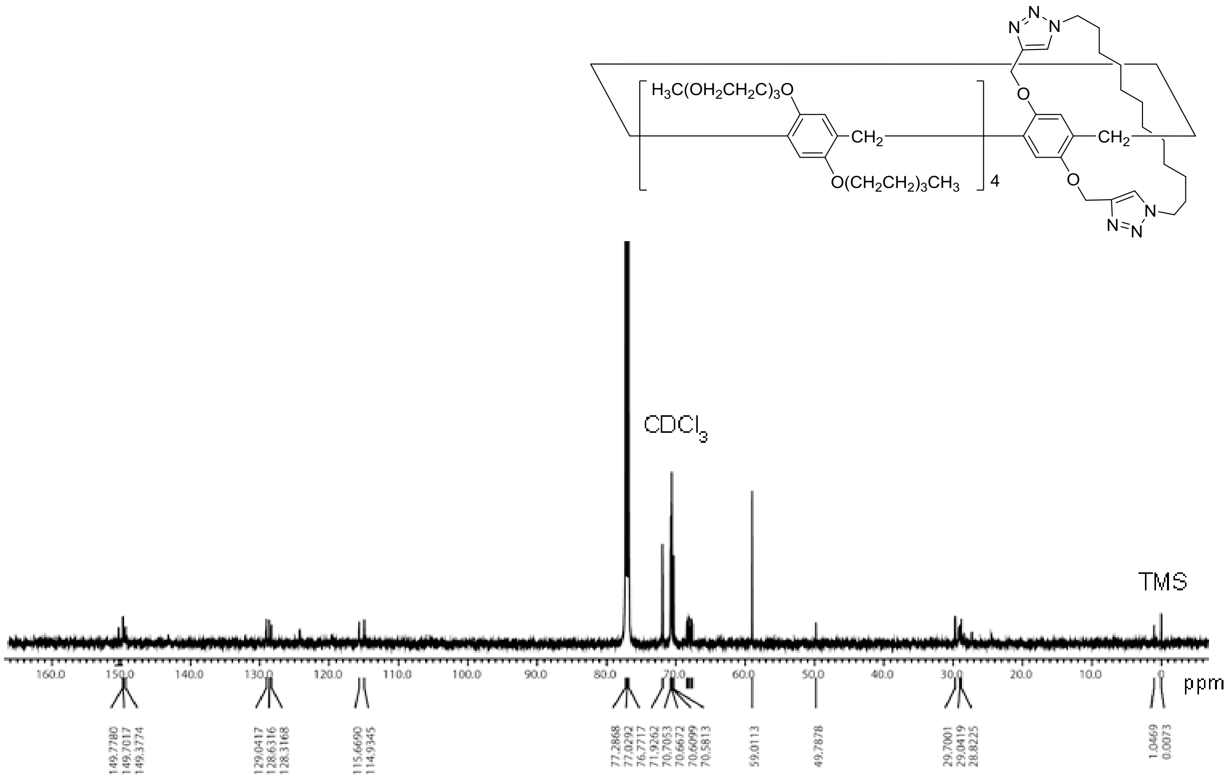


**Figure S4:** $^{13}$C NMR spectrum ($CDCl_3$, 25 °C) of **3**.

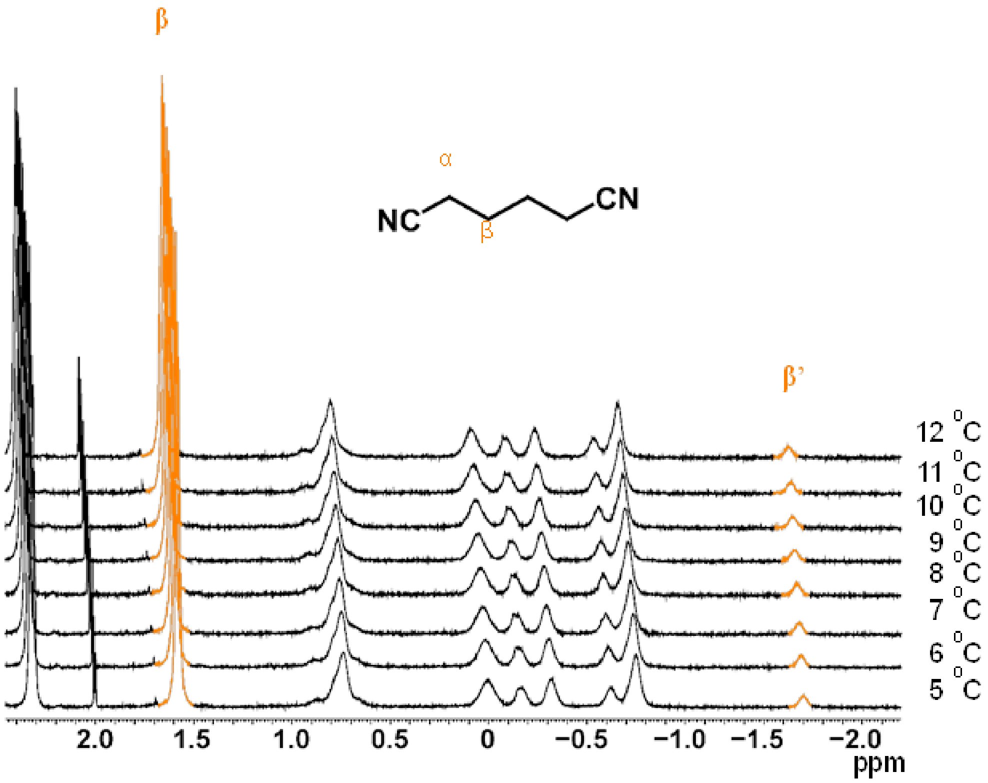


**Figure S5:** Variable temperature $^{1}$H NMR spectra ($D_2O$) of a mixture of **3** (2 mM) and 1,4-dicyanobutane (2 mM).

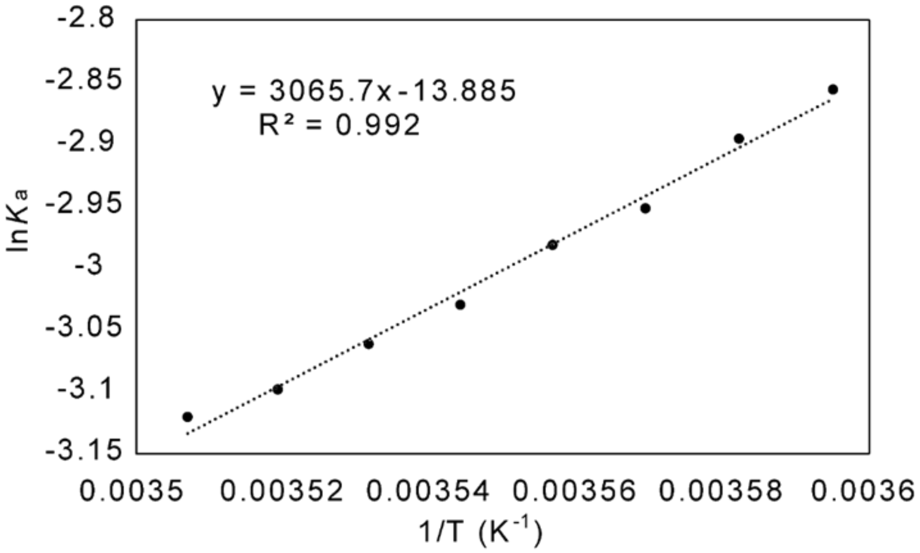


**Figure S6:** van't Hoff plots for a mixture of **3**-1,4-dicyanobutane complex.

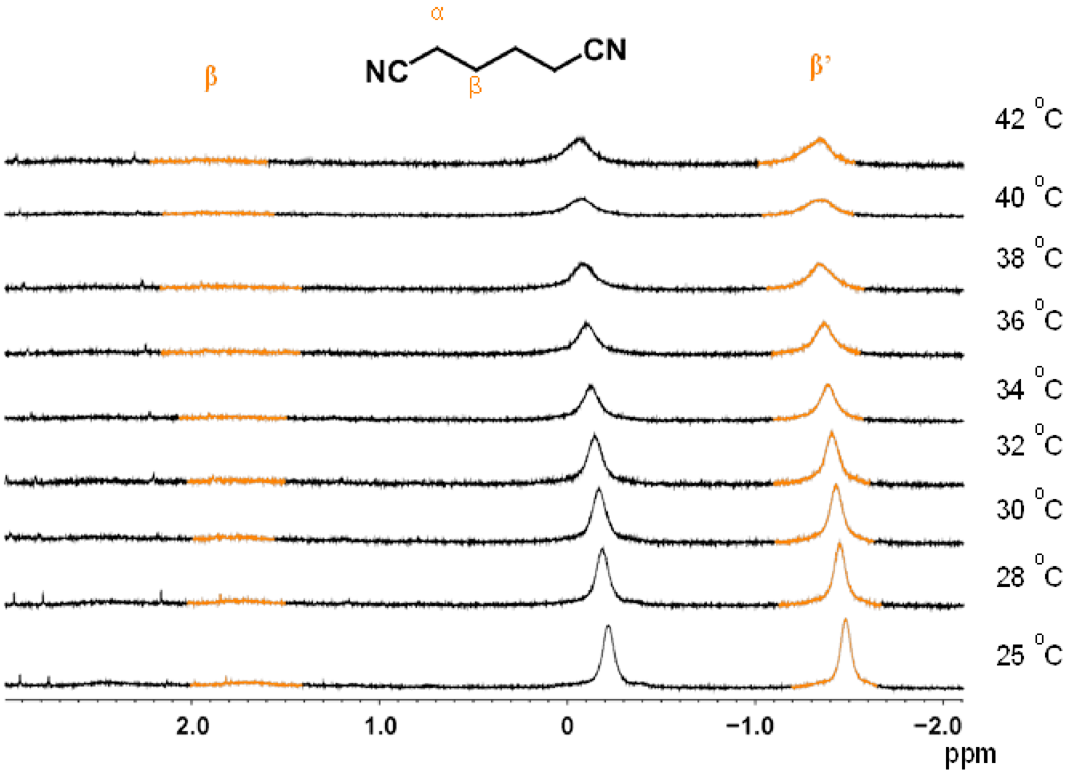


**Figure S7:** Variable temperature $^1$H NMR spectra ($D_2O$) of a mixture of **1** (2 mM) and 1,4-dicyanobutane (2 mM).

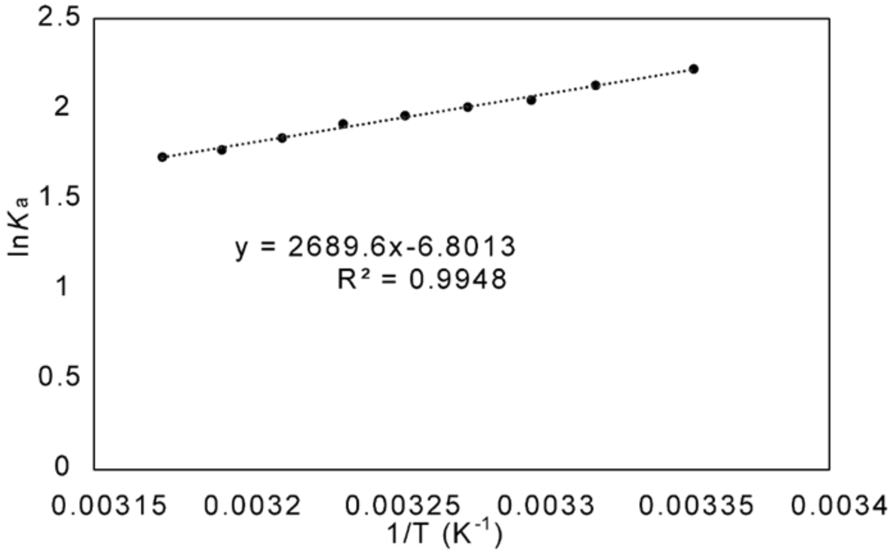


**Figure S8:** van’t Hoff plots for a mixture of **1**-1,4-dicyanobutane complex.
